# Supplementary material for: Salt-Related Knowledge, Attitudes, and Behaviors on Efate Island, Vanuatu
Source: Int J Environ Res Public Health. 2019 Mar 21;16(6):1027. doi: 10.3390/ijerph16061027 (PMC6466123; doi:10.3390/ijerph16061027)
Supplement: Supplementary file 1 [file ijerph-16-01027-s001.pdf]

Table S1. Knowledge, attitude and behavior questions relating to dietary salt intake (adapted from [1])

**Dietary Salt**	
The Next questions ask about your knowledge, attitudes and behaviour towards dietary salt. Dietary salt includes ordinary table salt, unrefined salt such as sea salt, iodized salt and salty sauces such as soya sauce or fish sauce (see show card). The following questions are on adding salt to the food right before you eat it, on how food is prepared in your home, on eating processed foods that are high in salt such as tin tuna, canned beef, noodles, twisties, and questions on controlling your salt intake. Please answer the questions even if you consider yourself to eat a diet low in salt.	
**Question**	**Response (Select only one)**
How often do you add salt to your food before you eat it or as you are eating it?<br>(USE SHOWCARD)	Always 1<br>Often 2<br>Sometimes 3<br>Rarely 4<br>Never 5<br>Don't know 77
How often is salt added in cooking or preparing foods in your household?<br>*(USE SHOWCARD)*	Always 1<br>Often 2<br>sometimes 3<br>Rarely 4<br>Never 5<br>Don't know 77
How often do you eat processed food high in salt such as breads, instant noodles, tinned and processed meats or sauces?<br>*(USE SHOWCARD)*	Always 1<br>Often 2<br>Sometimes 3<br>Rarely 4<br>Never 5<br>Don't know 77
**How much salt do you think you consume?**	Far too much 1<br>Too much 2<br>Just the right amount 3<br>Too little 4<br>Far too little 5<br>Don't know 77
Do you think that too much salt in your diet could cause a serious health problem?	Yes 1<br>No 2<br>Don't know 77
What do you think is the recommended amount of salt you should consume per day to be healthy?	Less than 10 g (2 teaspoon) 1<br>Less than 5 g (1 teaspoon) 2<br>Less than 2g (1/2 teaspoon) 3<br>Don't know 77
How important to you is lowering the salt in your diet	Very important 1<br>Somewhat important 2<br>Not at all important 3<br>Don't know 77

Do you do anything of the following on a regular basis to control your salt intake? *(RECORD FOR EACH)*	
Avoid/minimize consumption of processed foods	Yes 1<br>No 2
Look at the salt or sodium labels on food	Yes 1<br>No 2
Do not add salt on the table	Yes 1<br>No 2
Buy low salt/sodium alternatives	Yes 1<br>No 2
Do not add salt when cooking	Yes 1<br>No 2
Use spices other than salt when cooking	Yes 1<br>No 2
Avoid eating out	Yes 1<br>No 2
Other	Yes If yes, please specify<br>No 2
Other (please specify)	

Reference

1. World Health Organization. WHO STEPS Instrument (Core and Expanded_. Geneva, Switzerland: World Health Organization,; 2014.
